# Case Report: Acute liver injury with Epstein-Barr virus DNAemia following teplizumab: drug-induced liver injury, viral reactivation, or both?

**DOI:** 10.3389/fendo.2026.2008253

**Published:** 2026-10-29

**Authors:** Luca Zagaroli, Valentina Cattivera, Federica Guerra, Sabina Zara, Maria Laura Iezzi, Gabriella Bottone, Claudia Caloisi, Cecilia Fabiano, Cristina Gentile, Giulia Iapadre, Giuliana Nanni, Laura Palumbo, Vincenzo Salpietro, Maurizio Delvecchio

**Affiliations:** 1 Unit of Pediatrics, San Salvatore Hospital, L’Aquila, Italy; 2 Department of Biotechnological and Applied Clinical Sciences, University of L’Aquila, L’Aquila, Italy; 3 Dietetics Service, San Salvatore Hospital, L’Aquila, Italy

**Keywords:** case report, Epstein-Barr virus, liver injury, teplizumab, type 1 diabetes

## Abstract

**Background:**

Teplizumab (anti-CD3) delays progression from stage 2 to stage 3 type 1 diabetes (T1D) but can be associated with laboratory abnormalities and virologic findings, including Epstein-Barr virus (EBV) DNAemia.

**Methods:**

We report a 15-year-old boy with stage 2 T1D who received a standard course of teplizumab and developed a biphasic rise in aminotransferases occurring mainly after the end of infusions, with preserved bilirubin and coagulation parameters.

**Results:**

During treatment, ALT/AST rose to 78/44 U/L (1.90/1.10×ULN). After completion, ALT/AST increased to 292/172 U/L (7.12/4.30×ULN) on post-treatment day +5 and peaked at 428/220 U/L (10.44/5.50×ULN) on day +6, followed by partial improvement and a second flare beginning on day +20 and peaking on day +24 (ALT 293 U/L, 7.15×ULN; AST 118 U/L, 2.95×ULN; GGT 142 U/L), with normal bilirubin. Coagulation parameters remained within the normal range. Work-up for autoimmune hepatitis, hepatitis B/C, CMV, adenovirus, parvovirus B19, and toxoplasmosis was negative. Abdominal ultrasound/Doppler was unremarkable. MRI reported liver size mildly increased/at the upper limit of normal, nonspecific segment IV abnormalities, and reactive abdominal lymph nodes, with interval improvement at follow-up. Pre-treatment VCA IgG was positive and VCA IgM negative. At post-treatment day +10, whole-blood EBV DNA was 538,623 copies/mL, with negative VCA IgM, EA IgG and EBNA IgG, decreasing to <200 copies/mL at 6 months. Liver enzymes normalized spontaneously over follow-up.

**Conclusion:**

This case highlights marked, biphasic liver injury occurring after teplizumab in association with marked EBV DNAemia. The available clinical, serological, virological, and medication-timing data do not permit definitive attribution to teplizumab, EBV-related hepatic involvement, or concomitant medications. Post-treatment monitoring of both liver enzymes and EBV DNA may be helpful.

## Introduction

Teplizumab, a humanized anti-CD3 monoclonal antibody, is the first disease-modifying therapy approved to delay progression from stage 2 to stage 3 type 1 diabetes (T1D) (1, 2). Its immunomodulatory effects are associated with a safety profile that includes transient lymphopenia, cytokine release syndrome (CRS), and elevations in liver aminotransferases (1–3). Epstein-Barr virus (EBV) reactivation and DNAemia have also been reported following teplizumab, typically occurring within the first weeks after treatment and often in the absence of overt EBV-related disease (1–3).

The coexistence of liver-enzyme abnormalities and EBV DNAemia after anti-CD3 therapy creates a relevant diagnostic challenge. EBV can itself cause acute hepatitis, whereas teplizumab is associated with transient liver-enzyme elevations, either as part of CRS or potentially through other drug- or immune-mediated mechanisms (1, 3, 4). Distinguishing these possibilities is particularly difficult when EBV DNA is detected without a compatible clinical syndrome or characteristic serological evolution, because DNAemia alone does not establish EBV-related organ disease (5, 6).

We report a 15-year-old boy with stage 2 T1D who developed marked, biphasic liver injury and EBV DNAemia after a 14-day teplizumab course, without a mononucleosis-like syndrome. This case illustrates the difficulty of distinguishing drug- or immune-mediated liver injury from EBV-related hepatic involvement and highlights the importance of temporally aligned biochemical and virological monitoring.

## Methods

We reviewed the clinical, biochemical, virological, and glycemic data of a 15-year-old boy with stage 2 T1D treated with teplizumab. Pre-treatment eligibility was assessed according to the teplizumab criteria applicable at the time of treatment, including age ≥8 years, adequate bone-marrow function (hemoglobin ≥10 g/dL, lymphocyte count ≥1.0 × 10^9^/L, absolute neutrophil count ≥1.5×10^9^/L, and platelet count ≥150×10^9^/L), liver tests within the eligibility limits (ALT and AST ≤2×upper limit of normal, ULN, and total bilirubin ≤1.5×ULN), completion of age-appropriate vaccinations, and absence of active serious infection (7).

The patient was recruited at the Pediatric Unit. He was a 15-year-old boy, living with his parents, and diagnosed with stage 2 T1D after detection of impaired fasting glucose and positivity for multiple pancreatic islet autoantibodies (IA-2, ZnT8, and GAD65). At diagnosis, fasting plasma glucose was 115 mg/dL and HbA1c was 6.2%; an OGTT was not performed because two dysglycemic criteria were already present. Pre-treatment glycated hemoglobin was 6.1%. Physical examination was unremarkable, with no hepatomegaly, jaundice, or other relevant findings. At treatment initiation, weight was 62.5 kg, height 176.1 cm, BMI 20.2 kg/m², and documented body surface area was 1.74 m². The patient denied any tobacco or alcohol consumption. He was born prematurely at 36 weeks. The personal history was characterized by respiratory atopy and a comorbid emotional-behavioral disorder with average cognitive functioning. He exhibited unspecified developmental disorders of scholastic skills. Due to these neurodevelopmental challenges, an Individualized Education Program had been implemented in his school setting. His mother developed T1D at 19 years of age. His father and his 27-year-old sister had no relevant medical history.

At the time of treatment (July 2025), screening for acute EBV and CMV infection was based on clinical and laboratory assessment, including serology, rather than routine pre-treatment viral-load PCR, which was not required (7). Serial pre-treatment EBV serology showed VCA IgG positivity with negative VCA IgM and no serological evidence of acute EBV infection.

Teplizumab was administered intravenously according to the standard 14-day dosing schedule, based on documented BSA 1.74 m² (**Table 1**) (7). The 14-day course was completed. Absolute lymphocyte counts below 0.5×10³/µL on treatment days 5–7 met CTCAE grade 3 lymphopenia and subsequently recovered. Premedication 30 minutes before each infusion consisted of ibuprofen 400 mg and chlorphenamine 10 mg, administered daily from treatment day 1 through day 14. Other inpatient medications included pantoprazole, a single dose of paracetamol, cetirizine, and granisetron as needed. A midline catheter was used for intravenous administration. Clinical, biochemical, hematological, and virological data were reviewed from baseline to approximately 6 months after treatment. Liver-enzyme changes were assessed relative to teplizumab administration and expressed as multiples of the ULN. Whole-blood EBV DNA was interpreted alongside serology and clinical findings.

**Table 1:** Teplizumab infusion course: dose, lymphocyte count, C-reactive protein (CRP), aminotransferases (ALT; AST), and total bilirubin.

Treatment day	Teplizumab dose (µg)	Absolute lymphocyte count (x10^3^/µL)	CRP (mg/L)	ALT (U/L (×ULN))	AST (U/L (×ULN))	Total bilirubin (mg/dL)
Day 1	110	2.61	0.4	24 (0.59×)	20 (0.50×)	1.01
Day 2	220	2.02	7.2	23 (0.56×)	18 (0.45×)	0.73
Day 3	440	0.58	8.6	18 (0.44×)	13 (0.33×)	0.45
Day 4	880	0.56	6	20 (0.49×)	16 (0.40×)	0.47
Day 5	1800	0.31	7	21 (0.51×)	18 (0.45×)	0.67
Day 6	1800	0.35	10	21 (0.51×)	17 (0.43×)	0.52
Day 7	1800	0.45	8.2	26 (0.63×)	25 (0.63×)	0.35
Day 8	1800	0.78	4.7	44 (1.07×)	33 (0.83×)	0.59
Day 9	1800	0.95	2.6	62 (1.51×)	44 (1.10×)	0.48
Day 10	1800	1.02	1.5	71 (1.73×)	43 (1.08×)	0.57
Day 11	1800	1.29	1	78 (1.90×)	41 (1.03×)	0.31
Day 12	1800	1.03	0.7	66 (1.61×)	33 (0.83×)	0.37
Day 13	1800	0.93	0.8	65 (1.59×)	29 (0.73×)	0.53
Day 14	1800	0.60	0.6	50 (1.22×)	20 (0.50×)	0.57
Post-treatment day +1	–	0.70	0.6	40 (0.98×)	19 (0.48×)	0.34

Local reference values: absolute lymphocyte count ≥0.80x10^3^/µL; CRP <5 mg/L; ALT (ULN = 41 U/L); AST (ULN = 40 U/L). Total bilirubin reference range 0–1.2 mg/dL.

## Results

During the infusion course, laboratory monitoring showed transient grade 3 lymphopenia, with absolute lymphocyte counts falling from 2.61×10^3^/µL at baseline to a nadir of 0.31×10^3^/µL on treatment day 5, followed by gradual recovery. C-reactive protein increased modestly (peak 10 mg/L on treatment day 6). Aminotransferases rose mildly during treatment (ALT peak 78 U/L (1.90×ULN) on treatment day 11; AST peak 44 U/L (1.10×ULN) on treatment day 9) without bilirubin increase. The patient was discharged at the end of the 14-day treatment. Daily blood counts, inflammatory markers, and liver biochemistry are summarized in **Table 1**.

Incidentally, a non-occlusive catheter-associated thrombosis of the right axillary/subclavian venous axis was identified on treatment day 14 (6 August 2025) by Doppler ultrasound performed before midline removal, without clinical signs of limb ischemia. Therapeutic low-molecular-weight heparin (6000 IU every 12 h) was started on the same day and continued until the switch to rivaroxaban, and the midline catheter was planned for removal after anticoagulation, per vascular surgery recommendations. After the improvement in liver enzymes, anticoagulation was transitioned to rivaroxaban 20 mg/day (post-treatment day +32) and continued until 3 months from deep vein thrombosis diagnosis.

## Post-treatment liver injury and diagnostic work-up

After completion of teplizumab infusions, aminotransferases increased markedly with a biphasic pattern (**Table 2**). The overall clinical, laboratory, virologic, and imaging timeline is summarized in **Figure 1**. The first flare began on post-treatment day +5 (ALT 292 U/L, 7.12×ULN; AST 172 U/L, 4.30×ULN) and peaked on post-treatment day +6 (ALT 428 U/L, 10.44×ULN; AST 220 U/L, 5.50×ULN), with persistently normal total bilirubin and INR, and CRP consistently <5 mg/L. Aminotransferases partially decreased over subsequent days (ALT 417 U/L (10.17×ULN) on post-treatment day +7 and 384 U/L (9.37×ULN) on day +10), followed by a second flare with a concomitant increase in GGT (day +20: ALT 251 U/L (6.12×ULN), AST 136 U/L (3.40×ULN), GGT 92 U/L; day +24: ALT 293 U/L (7.15×ULN), AST 118 U/L (2.95×ULN), GGT 142 U/L), again with normal bilirubin and preserved coagulation. Alkaline phosphatase (ALP) was not measured at the day +5/+6 peak; on day +7 ALP was 192 U/L (local laboratory ULN 150 U/L), giving a descriptive R ratio of approximately 8.0, while on day +24 ALP was 268 U/L with an R ratio of approximately 4.0. Because the local ALP reference interval was not age- and sex-adjusted, these R ratios were interpreted cautiously. The temporal trends of ALT, AST, GGT, and whole-blood EBV DNA are shown in **Figure 2**. Values subsequently improved (ALT 240 U/L (5.85×ULN) on post-treatment day +28; ALT 59 U/L (1.44×ULN) on post-treatment day +35) and normalized on post-treatment day +56 (ALT 34 U/L (0.83×ULN); AST 27 U/L (0.68×ULN); GGT 22 U/L), with stable values thereafter. During the transaminase rise the patient was asymptomatic. Autoimmune liver testing showed negative ANA, SMA/ASMA, anti-LKM/anti-LKM1, anti-SLA and AMA/M2-related antibodies (liver-dot specificities <6 AU); total IgG was not measured during the acute flare and was normal at later follow-up (1122 mg/dL). Hepatitis B and C, CMV, adenovirus, parvovirus B19, and toxoplasmosis were negative. HAV, HEV, HSV, VZV, HHV-6 and ceruloplasmin were not assessed during the acute episode. Tissue-transglutaminase IgA was 2.3 U/mL with normal total IgA (191 mg/dL), creatine kinase was 80 U/L, and alpha-1 antitrypsin was 160.4 mg/dL. Abdominal ultrasound with Doppler showed no biliary obstruction or vascular thrombosis. MRI on post-treatment day +57 reported liver size mildly increased/at the upper limit of normal, subtle nonspecific subcapsular signal abnormalities in segment IV, and mildly enlarged reactive upper-abdominal lymph nodes, without biliary abnormalities. Hepatic steatosis was not reported, and quantitative proton-density fat fraction was not performed. At approximately 6 months, imaging showed partial regression of these abnormalities. At approximately 6 months, total bilirubin was 0.72 mg/dL and remained within the normal reference range. Secondary HLH was considered: the patient remained afebrile, triglycerides were 94 mg/dL and fibrinogen 249 mg/dL; ferritin and soluble IL-2 receptor were not measured during the acute flare, so complete retrospective exclusion according to HLH criteria was not possible.

**Table 2:** Post-teplizumab liver biochemistry, EBV studies, and key investigations.

ALT (U/L)	AST (U/L)	ALP (U/L)	GGT (U/L)	Timepoint	Total bilirubin, mg/dL	INR	CRP, mg/L	EBV studies	Key investigations/comments
–	–	–	–	10 weeks before treatment	–	–	–	Anti-EBV VCA IgG 59.5 U/mL; anti-EBV VCA IgM <10.0 U/mL	Baseline serology showed pre-existing VCA IgG positivity
–	–	–	–	Pre-treatment	–	–	–	Anti-EBV VCA IgG 82.8 U/mL; anti-EBV VCA IgM <10.0 U/mL	Immediate pre-treatment serology
	–	–	–	Post-treatment day +2	–	–	–	–	No blood sample obtained
	–	–	–	Post-treatment day +3	–	–	–	–	No blood sample obtained
	–	–	–	Post-treatment day +4	–	–	–	–	No blood sample obtained
292	172	–	20	Post-treatment day +5	–	–	0.05	–	First major flare Abdominal ultrasound with Doppler: no biliary obstruction or vascular thrombosis
428	220	–	22	Post-treatment day +6	0.40	–	–	–	Peak ALT/AST
417	172	192	23	Post-treatment day +7	0.36	1.08	0.04	Anti-EBV VCA IgG 83.7 U/mL; anti-EBV VCA IgM <10.0 U/mL; anti-EBV EA IgG <5.00 U/mL; anti-EBV EBNA IgG <3.00 U/mL	Acute-phase EBV serology
384	138	–	27	Post-treatment day +10	0.35	–	0.04	Whole-blood EBV DNA 538,623 copies/mL	ALC 1.35 ×10^3^/µL
275	72	–	29	Post-treatment day +12	–	–	0.04	–	Partial improvement
165	57	–	30	Post-treatment day +16	–	1.07	0.18	–	Persistent but improving hepatocellular pattern
251	136	–	92	Post-treatment day +20	–	–	–	–	Second flare with more evident cholestatic-enzyme component
293	118	268	142	Post-treatment day +24	0.37	–	0.22	–	Second peak GGT; LDH 308 U/L
240	86	237	122	Post-treatment day +28	–	–	0.11	–	LDH 351 U/L
59	27	–	58	Post-treatment day +35	0.50	1.19	0.04	–	Near normalization
34	27	209	22	Post-treatment day +56	0.47	1.04	–	–	Normalization
				Post-treatment day +57					Liver MRI: Mild hepatomegaly with relative right-lobe hypertrophy; subtle subcapsular signal alterations in segment IV; spleen at upper limit of normal; mildly enlarged reactive upper-abdominal lymph nodes (including porta hepatis nodes up to 10 × 25 mm and a left para-aortic node up to 7 × 15 mm); no biliary dilatation.
29	26	245	7	Post-treatment day +105	0.44	1.14	0.04	–	Stable liver tests at follow-up
–	–	–	–	Approximately day +180 follow-up	0.72	–	–	Anti-EBV VCA IgG 676 U/mL; anti-EBV VCA IgM <10.0 U/mL; whole-blood EBV DNA below the laboratory reporting cut-off	LDH 191 U/L Liver MRI: segment IV abnormalities smaller and less conspicuous; reduced splenic bipolar diameter 11.2 cm vs 12 cm; persistent liver size at upper limit of normal (right-lobe longitudinal diameter 18.5 cm); stable hilar hepatic and lumbo-aortic retroperitoneal lymph nodes; no free fluid
–	–	–	–	Etiologic work-up	–	–	–	CMV and adenovirus serologies negative; parvovirus B19 DNA negative; toxoplasma confirmatory testing negative	Autoimmune hepatitis work-up negative; HBsAg and anti-HCV negative

Post-treatment day numbering is calculated from the last teplizumab infusion. Whole-blood EBV DNA was quantified using the quanty EBV real-time PCR assay (Clonit, Italy; RT-13), a CE-IVD-marked assay targeting the BamHI-W region of the EBV genome, on a QIAGEN platform. The elevated EBV DNA result was confirmed by repeat testing. EBV DNA and ALT measurements on post-treatment day +10 were performed on samples obtained during the same blood collection. Results were reported in copies/mL. ALC, absolute lymphocyte count; CRP, C-reactive protein; INR, international normalized ratio; EBV, Epstein-Barr virus; VCA, viral capsid antigen; EA, early antigen; EBNA, Epstein-Barr nuclear antigen; DNA, deoxyribonucleic acid; LDH, lactate dehydrogenase; ALP, alkaline phosphatase; GGT, gamma-glutamyl transferase; MRI, magnetic resonance imaging; CMV, cytomegalovirus; HBsAg, hepatitis B surface antigen; anti-HCV, hepatitis C virus antibody. Local EBV assay cut-offs: VCA IgG <20 U/mL negative and ≥20 U/mL positive; VCA IgM <20 U/mL negative, 20–40 U/mL borderline, and >40 U/mL positive; EA IgG <10 U/mL negative, 10–40 U/mL borderline, and >40 U/mL positive; EBNA IgG <5 U/mL negative, 5–20 U/mL borderline, and >20 U/mL positive; whole-blood EBV DNA <200 copies/mL below the assay cut-off. Local reference limits used in the table: ALT ULN 41 U/L; AST ULN 40 U/L; GGT ULN 64 U/L; ALP 40–150 U/L (not age- and sex-adjusted); LDH ULN 220 U/L.

**Figure 1:**
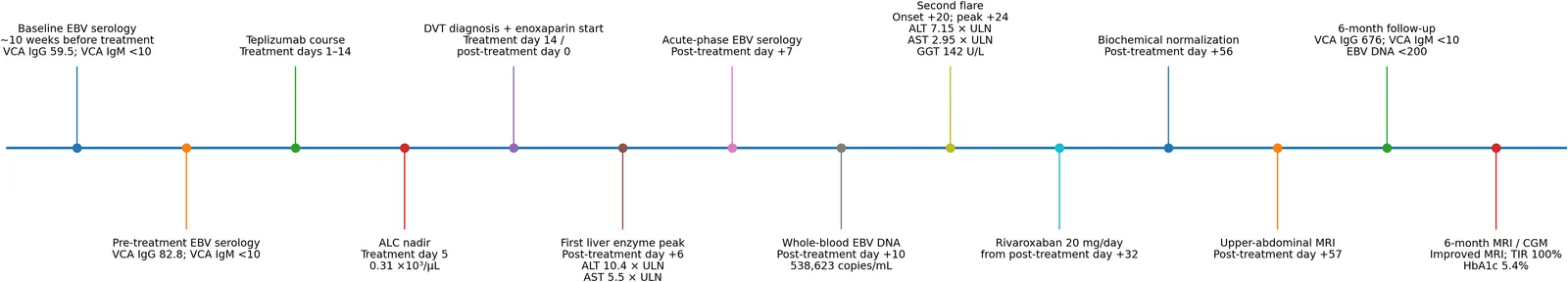
Clinical timeline of teplizumab treatment, lymphocyte nadir, liver-enzyme flares, catheter-associated thrombosis and anticoagulation, EBV testing, imaging, and follow-up.

**Figure 2:**
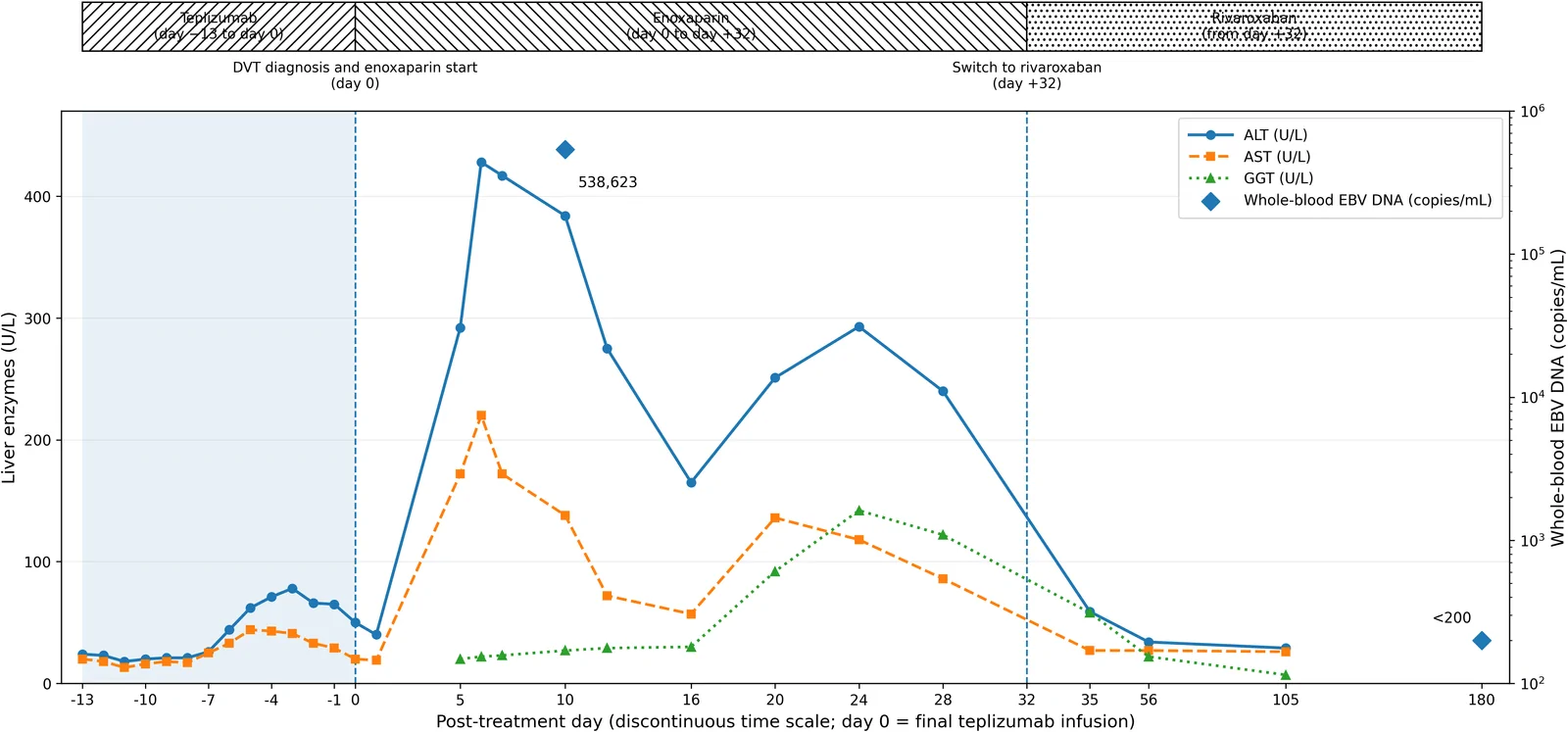
Temporal trends of ALT, AST, GGT, and whole-blood EBV DNA during and after teplizumab treatment. The shaded area indicates the teplizumab treatment period. ALT, AST, and GGT are expressed in U/L (left y-axis), whereas EBV DNA is expressed in copies/mL (right y-axis, logarithmic scale). The upper limits of normal were 41 U/L for ALT, 40 U/L for AST, and 64 U/L for GGT. The x-axis indicates post-treatment days, with day 0 corresponding to the final teplizumab infusion. No blood samples were obtained on post-treatment days +2 to +4; connecting lines between measured points are for graphical representation only. The EBV DNA value at day +180 was below the assay reporting cut-off (<200 copies/mL) and is plotted at 200 copies/mL for visualization purposes.

## EBV testing

Pre-treatment EBV serology showed anti-EBV VCA IgG 82.8 U/mL (reference: ≥20 positive) and anti-EBV VCA IgM <10.0 U/mL (reference: <20 negative). In the acute phase, anti-EBV VCA IgG was 83.7 U/mL, showing no major change from baseline, while anti-EBV VCA IgM remained <10.0 U/mL. Anti-EBV EA IgG was <5.00 U/mL (reference: <10 negative) and anti-EBV EBNA IgG was <3.00 U/mL (reference: <5 negative). Whole-blood EBV PCR was positive at 538,623 copies/mL (assay cut-off <200 copies/mL) on post-treatment day +10. EBNA IgG was not measured before treatment or at 6 months, and VCA IgG avidity testing was not available. Plasma/serum EBV DNA, lymphocyte subsets, and a peripheral-blood smear for atypical lymphocytes were not assessed. At 6-month follow-up, anti-EBV VCA IgG remained positive and further increased to 676 U/mL, anti-EBV VCA IgM remained negative at <10.0 U/mL, and whole-blood EBV DNA decreased to <200 copies/mL, below the assay cut-off. Clinically, the patient did not develop mononucleosis syndrome (no fever, pharyngitis, or clinically significant lymphadenopathy), and liver synthetic function remained preserved throughout.

## Glycemic outcomes

Continuous glucose monitoring showed that time in range increased from 97% at pre-treatment to 100% at follow-up. No time was spent in hypoglycemia at either assessment. HbA1c decreased from 6.1% pre-treatment to 5.4% at 6 months and 5.5% at 13 months, when the patient was still in stage 2 type 1 diabetes.

## Discussion

This case describes biphasic liver injury after teplizumab, with preserved bilirubin and coagulation and concurrent EBV DNAemia. The temporal pattern raises the possibility of drug- or immune-mediated injury and EBV-related hepatic involvement. Enoxaparin was also considered as a potential contributor. Although low-molecular-weight heparins can cause transient asymptomatic aminotransferase elevations (8), the initial mild liver-enzyme abnormalities preceded enoxaparin and therefore cannot be attributed to it. However, enoxaparin was started on treatment day 14, corresponding to post-treatment day 0 and thus a contribution to the subsequent post-treatment flares cannot be excluded. Daily ibuprofen overlapped the onset of the aminotransferase increase, whereas rivaroxaban began only on post-treatment day +32, after both flare peaks. The updated RUCAM framework was considered, but a numerical score was not regarded as reliable because ALP was unavailable at the first peak and several temporally overlapping candidate causes were present.

Transient liver-enzyme abnormalities are a recognized adverse effect of teplizumab, but the pattern observed in our patient appears unusual. Clinical trials and regulatory data indicate that aminotransferase elevations are generally early, transient, and self-limited, and may occur both with and without CRS (1, 3, 9) (**Supplementary Table S1**). When associated with CRS, liver-test abnormalities typically occur during the first days of treatment. Recent evidence is consistent with this pattern. In the PETITE-T1D study, early aminotransferase elevation leading to treatment discontinuation resolved within days (10), while in a 2026 Italian real-world series, one of three adults receiving teplizumab developed mild, transient aminotransferase elevation with lymphopenia that resolved spontaneously (11). A larger prospective real-world study of teplizumab-treated individuals with stage 2 T1D similarly reported a safety profile broadly consistent with previous clinical trials (12). In contrast, although our patient showed a mild increase during treatment, the major aminotransferase peak occurred 6 days after completion of the 14-day course, in the absence of clinical CRS, and was followed by a second biochemical flare. The magnitude, delayed peak, and biphasic trajectory therefore differ from the more commonly reported hepatic safety signal of teplizumab. In addition, the on-treatment aminotransferase rise became evident on treatment days 8–11 without fever, hypotension, or other clinical features of CRS; no persistent systemic pattern suggestive of a serum-sickness-like reaction was observed.

EBV reactivation and DNAemia are recognized phenomena following immunomodulatory therapy, including anti-CD3 treatment with otelixizumab (13). Available evidence indicates that transient perturbation of cellular immune control may allow reactivation of latent EBV and detectable EBV DNAemia without clinically overt EBV disease. Following teplizumab, EBV DNAemia typically emerges within weeks and is predominantly asymptomatic and self-limited, even at high viral loads (1; **Supplementary Table S1**). Thus, EBV DNAemia may reflect transient impairment of EBV immune control rather than clinically significant EBV disease.

These observations are directly relevant to our patient, in whom whole-blood EBV DNA reached 538,623 copies/mL on post-treatment day +10, within the temporal window previously described for post-teplizumab EBV DNAemia, but without fever, pharyngitis, clinically relevant lymphadenopathy, or other manifestations of a mononucleosis-like syndrome. Moreover, EBV viral-load measurements are assay- and compartment-dependent: whole-blood measurements include cell-associated EBV DNA and are not interchangeable with plasma or serum measurements (5, 6). Thus, marked whole-blood DNAemia alone does not establish EBV hepatitis and must be interpreted alongside clinical, serological, and biochemical findings (6).

The serological findings in our patient further illustrate this uncertainty. Serial pre-treatment testing demonstrated VCA IgG positivity with persistently negative VCA IgM, while EBNA IgG was not measured before treatment, so a relatively recent primary infection could not be excluded solely from this pattern. During the acute liver-enzyme elevation, VCA IgG remained essentially unchanged, while VCA IgM, EA IgG, and EBNA IgG were negative despite marked EBV DNAemia. Serological patterns during EBV reactivation may be atypical, particularly under immunomodulatory conditions, and do not necessarily parallel viral DNA kinetics (5, 6). The absence of a mononucleosis-like syndrome and negative VCA IgM/EA IgG cannot, however, exclude EBV-related hepatic involvement in an anti-CD3-treated, lymphopenic patient. The subsequent increase in VCA IgG to 676 U/mL may indicate evolving humoral exposure but does not establish the timing or cause of the hepatic abnormality.

Klemann and Kordonouri reported fulminant EBV reactivation with secondary hemophagocytic lymphohistiocytosis and multiorgan involvement after teplizumab in an adult with trisomy 21, with EBV DNA exceeding 2 million copies/mL and supportive systemic and histopathological findings (14). This phenotype contrasts markedly with our patient, who remained clinically well despite marked EBV DNAemia and substantial aminotransferase elevation. In our patient, the absence of fever and the normal triglyceride and fibrinogen values argued against an overt HLH phenotype, although ferritin and soluble IL-2 receptor were not measured during the acute flare. These cases illustrate the spectrum of EBV-associated findings after teplizumab, from asymptomatic DNAemia to severe systemic disease.

Causal attribution in our patient therefore remains uncertain. The first major aminotransferase peak occurred on post-treatment day +6. The EBV DNA sample was obtained on post-treatment day +10, during the descending phase of the first flare, when ALT remained 384 U/L (9.37×ULN). No pre-treatment EBV DNA, no viral-load measurement at the day +5/+6 peak, and no viral-load measurement during the second flare were available. Although this timing is compatible with the recognized window for post-teplizumab EBV DNAemia, a single viral-load measurement cannot establish whether viral replication preceded, coincided with, or followed the hepatic injury. Conversely, the onset of liver-enzyme abnormalities during teplizumab exposure, followed by a marked post-treatment peak, a second flare, preserved bilirubin and INR, and spontaneous biochemical recovery supports a possible drug- or immune-mediated contribution. Accordingly, the available data do not permit reliable distinction among teplizumab-associated or immune-mediated liver injury, EBV-related hepatic involvement, and concomitant medication effects.

This case also has implications for clinical monitoring. At the time our patient was treated in July 2025, screening for acute EBV and CMV infection was based on clinical and laboratory assessment, including serology, and routine pre-treatment viral-load PCR was not required (7). Regulatory and expert recommendations have subsequently evolved. The 2026 BSPED/ABCD Consensus Statement recommends screening for active viral infections, including EBV and CMV, within 4 weeks before teplizumab initiation and emphasizes interpretation of virological results in the clinical context (15). Current US prescribing information has similarly strengthened recommendations regarding assessment and monitoring for viral reactivation (16). Health Canada also issued a Health Professional Risk Communication on 14 September 2026 addressing viral reactivation with teplizumab (17). These developments reinforce the clinical relevance of the present case. When significant liver-enzyme abnormalities occur after teplizumab, serial and temporally aligned assessment of aminotransferases and EBV DNA may help distinguish treatment-associated DNAemia from clinically relevant EBV disease and improve causal attribution. Based on this case, an experience-based approach could include weekly ALT, AST, ALP, GGT and bilirubin during the first 4 weeks after the last infusion, with EBV/CMV PCR at approximately 2–4 weeks and earlier repeat testing if liver-enzyme abnormalities or symptoms suggest viral reactivation; clinical surveillance should continue for at least 2 months.

## Strengths and limitations

Strengths of this report include detailed longitudinal biochemical monitoring, serial pre-treatment EBV serology, whole-blood EBV DNA assessment during the acute phase, and extended clinical, virological, and imaging follow-up. The main limitation is the absence of EBV DNA measurements before treatment, at the day +5/+6 aminotransferase peak, and during the second flare, as well as the lack of parallel plasma/serum testing. These gaps preclude reliable temporal attribution of liver injury to EBV. Concomitant ibuprofen and enoxaparin are additional potential confounders; although the initial mild aminotransferase abnormalities preceded enoxaparin, the subsequent marked flares overlapped anticoagulation. ALP was not measured at the first peak, and HAV, HEV, HSV, VZV, HHV-6 and ceruloplasmin were not assessed during the acute episode. Finally, as this is a single case, causality cannot be established and the findings may not be generalizable to other teplizumab-treated patients.

## Conclusions

In this patient, marked, biphasic liver-enzyme elevation occurred predominantly after teplizumab treatment, accompanied by substantial EBV DNAemia. The available clinical, biochemical, and virological findings do not permit definitive attribution to teplizumab, EBV-related hepatic involvement, or concomitant medications, including enoxaparin and ibuprofen. Despite marked aminotransferase elevation, bilirubin and coagulation remained preserved, and liver abnormalities resolved spontaneously.

## Patient perspective

Before starting teplizumab, I was hopeful that the treatment could delay the onset of T1D, but I was also worried about possible side effects. The treatment required hospitalization and intravenous infusions. During and after treatment, some unexpected medical problems occurred, requiring additional tests and treatment. This was concerning. However, knowing that the healthcare team was closely following my condition and explaining what was happening helped me feel safer and more confident. Over time, these problems resolved, and I returned to feeling well. Despite these difficulties, I still view the treatment positively because of the possibility of delaying diabetes. I think that patients should be clearly informed not only about the expected benefits, but also about possible side effects and the importance of careful monitoring during and after treatment.

## Data Availability

The raw data supporting the conclusions of this article will be made available by the authors, without undue reservation.
