# Supplementary material for: Case Report: Acute liver injury with Epstein-Barr virus DNAemia following teplizumab: drug-induced liver injury, viral reactivation, or both?
Source: Front Endocrinol (Lausanne). 2026 Oct 29;17:2008253. doi: 10.3389/fendo.2026.2008253 (PMC13760551; doi:10.3389/fendo.2026.2008253)
Supplement: Supplementary file 1 [file SupplementaryFile1.docx]

**CARE Checklist – Teplizumab Case Report**

**Manuscript assessed:** Case report: Acute Liver Injury With Epstein–Barr Virus DNAemia Following Teplizumab: Drug-Induced Liver Injury, Viral Reactivation, or Both?

**Checklist:** CARE 2013.

| **Topic** | **Item** | **CARE requirement** | **Reported on** | **Assessment** |
| --- | --- | --- | --- | --- |
| Title | 1 | The diagnosis or primary intervention followed by “case report”. | Title | Complete |
| Keywords | 2 | 2–5 key words identifying diagnoses/interventions, including “case report”. | Keywords | Complete |
| Abstract | 3a | What is unique about the case and what it adds to the literature. | Abstract | Complete |
| Abstract | 3b | Main symptoms and/or important clinical findings. | Abstract | Complete |
| Abstract | 3c | Main diagnoses, therapeutic interventions, and outcomes. | Abstract | Complete |
| Abstract | 3d | Main take-away lesson. | Abstract | Complete |
| Introduction | 4 | Brief background explaining why the case is unique/relevant, with references. | Introduction | Complete |
| Patient information | 5a | De-identified patient-specific information. | Methods – Patient information | Complete |
| Patient information | 5b | Primary concerns and symptoms. | Methods; Results | Complete – stage 2 T1D was asymptomatic; the patient also remained asymptomatic during liver injury |
| Patient information | 5c | Medical, family, and psychosocial history, including relevant genetic information. | Methods – Patient information | Complete – personal history, neurodevelopmental history and family history are reported |
| Patient information | 5d | Relevant past interventions and their outcomes. | Methods – Patient information | N/A / no relevant prior intervention identified |
| Clinical findings | 6 | Relevant physical examination and other significant clinical findings. | Methods – Patient information | Complete – normal examination; absence of hepatomegaly/jaundice and other relevant findings stated |
| Timeline | 7 | Important information from the episode of care organized as a timeline. | Figure 1 | Complete |
| Diagnostic assessment | 8a | Diagnostic methods (physical examination, laboratory testing, imaging, surveys). | Methods; Results | Complete |
| Diagnostic assessment | 8b | Diagnostic challenges. | Results; Discussion | Complete – uncertainty in distinguishing drug-associated injury from EBV-related disease |
| Diagnostic assessment | 8c | Diagnosis and differential diagnoses considered. | Results; Discussion | Complete |
| Diagnostic assessment | 8d | Prognostic characteristics when applicable. | Not applicable | N/A |
| Therapeutic intervention | 9a | Types of therapeutic intervention. | Methods; Results | Complete |
| Therapeutic intervention | 9b | Administration, dose, strength, and duration. | Methods; Table 1 | Complete – standard 14-day teplizumab regimen and premedication are described |
| Therapeutic intervention | 9c | Changes in therapeutic interventions and rationale. | Results | Complete – LMWH followed by rivaroxaban for catheter-associated thrombosis |
| Follow-up/outcomes | 10a | Clinician- and patient-assessed outcomes. | Results; Patient Perspective | Complete – biochemical/clinical outcomes plus Patient Perspective |
| Follow-up/outcomes | 10b | Important follow-up diagnostic and other test results. | Results; Table 2 | Complete – serial liver tests, EBV testing and imaging follow-up |
| Follow-up/outcomes | 10c | Intervention adherence and tolerability and how assessed. | Methods; Results | Complete – treatment stated to be well tolerated and completed |
| Follow-up/outcomes | 10d | Adverse and unanticipated events. | Results | Complete – liver injury, transient lymphopenia and catheter-associated thrombosis described |
| Discussion | 11a | Strengths and limitations of the case report. | Discussion | Complete |
| Discussion | 11b | Relevant medical literature. | Discussion | Complete |
| Discussion | 11c | Scientific rationale for conclusions, including possible causes. | Discussion | Complete – competing causal explanations and temporal relationships are discussed |
| Discussion | 11d | Primary take-away lessons in a one-paragraph conclusion. | Discussion; Conclusion | Complete |
| Patient perspective | 12 | Patient shares perspective or experience whenever possible. | Patient Perspective | Complete |
| Informed consent | 13 | Informed consent for publication. | Ethics Statement | Complete – written informed consent from legal guardians reported |
