# Supplementary material for: Case Report: Acute liver injury with Epstein-Barr virus DNAemia following teplizumab: drug-induced liver injury, viral reactivation, or both?
Source: Front Endocrinol (Lausanne). 2026 Oct 29;17:2008253. doi: 10.3389/fendo.2026.2008253 (PMC13760551; doi:10.3389/fendo.2026.2008253)
Supplement: Supplementary file 2 [file Table1.docx]

**Supplementary Table S1. Published evidence on liver abnormalities and Epstein–Barr virus findings following teplizumab**

| **Study/source** | **Population and treatment** | **Liver-related findings** | **EBV-related findings** | **Clinical EBV manifestations** | **Relevance to the present case** |
| --- | --- | --- | --- | --- | --- |
| **FDA Multidiscipline Review (1)** | Regulatory review of teplizumab clinical development program | Aminotransferase elevations recognized during treatment; liver-function monitoring and treatment-discontinuation criteria established | EBV reactivation events were more frequent with teplizumab than control (4.5% vs 2.3%). EBV DNA levels >1,000,000 copies/mL were observed in individual patients | Most patients with EBV DNAemia were asymptomatic and events were generally self-limited | Demonstrates that marked EBV DNAemia after teplizumab does not necessarily indicate clinically significant EBV disease |
| **Herold et al., TN-10 (2)** | Stage 2 T1D; teplizumab vs placebo | No biphasic post-treatment liver-injury phenotype comparable to the present case was specifically described | Quantifiable whole-blood EBV DNA detected in 8/44 (18.2%) teplizumab-treated participants vs 0/32 placebo; typically during weeks 3–6 | Only one participant reported mild upper-respiratory symptoms; most DNAemia was clinically silent | Establishes the typical timing and frequently asymptomatic nature of post-teplizumab EBV DNAemia |
| **Herold et al., integrated safety analysis (3)** | Stage 2 and stage 3 T1D trials; teplizumab n=773, control n=245 | Transient liver-test abnormalities recognized within the overall safety profile | EBV reactivation: 5.1% vs 2.4%; EBV viremia: 3.2% vs 1.2% in teplizumab vs control; usually detected 2–3 weeks after dosing | Generally asymptomatic and spontaneously resolving without antiviral treatment | Supports EBV DNAemia as a recognized treatment-associated phenomenon that does not, by itself, establish EBV-related organ disease |
| **Ramos et al., PROTECT (4)** | Children and adolescents with newly diagnosed stage 3 T1D; teplizumab vs placebo | Protocol-specified liver-function test elevations leading to treatment discontinuation occurred in 4.1% vs 0.9% with placebo | Transient EBV reactivation detected in 8 teplizumab-treated participants | Six of eight were asymptomatic; all EBV-related events resolved without antiviral therapy | Pediatric evidence showing that both EBV reactivation and liver-test abnormalities may occur after teplizumab without establishing a causal relationship between them |
| **EMA regulatory assessment/SmPC (5)** | Pooled regulatory clinical-trial safety data | Peak ALT >3×ULN in 7.8% and AST >3×ULN in 5.3%; most elevations were transient and resolved within 1–2 weeks. CRS-associated abnormalities generally occurred early | EBV reactivation recognized within the safety profile | Usually transient/self-limited | Highlights the atypical timing of the present case, characterized by a major post-treatment peak followed by a second biochemical flare |
| **PETITE-T1D (6)** | Children aged <8 years with stage 2 T1D; single-arm teplizumab study, n=23 | One participant discontinued on day 2 because of early ALT/AST elevation considered probably drug-related, with normalization within days | One child was excluded at screening because of asymptomatic EBV infection | No severe EBV-related syndrome reported among treated participants | Recent pediatric evidence of an early, transient teplizumab-associated liver-enzyme elevation, contrasting with the delayed biphasic course in the present case |
| **Guarnotta et al. (7)** | Three adults with stage 2 T1D receiving a 14-day teplizumab course in an Italian real-world compassionate-use program | One patient developed mild, transient transaminase elevation associated with lymphopenia, resolving spontaneously | No clinically relevant EBV-related organ disease reported | None reported | Real-world evidence further supports a generally mild and transient hepatic safety signal, contrasting with the magnitude and biphasic pattern observed in the present case |
| **Karakus et al. (8)** | Prospective real-world cohort of stage 2 T1D; teplizumab-treated n=30, untreated comparator n=10 | Liver-function test elevations were reported in 40% of teplizumab-treated participants; adverse events were generally mild, and no serious adverse events were reported. | EBV-specific TCRβ clonotypes were assessed longitudinally and were similar before and after teplizumab treatment; EBV viral-load reactivation was not directly assessed | No EBV-related clinical syndrome was specifically reported | Provides prospective real-world safety data showing that liver-test elevations may occur during routine teplizumab use, while not providing evidence that such abnormalities reflect clinically significant EBV disease |
| **Klemann and Kordonouri (9)** | 44-year-old adult with trisomy 21 and stage 2 T1D following a standard 14-day teplizumab course | Six days after treatment completion, hepatic dysfunction occurred as part of severe multiorgan disease | Marked EBV reactivation with EBV PCR >2,000,000; additional evidence supported EBV-associated lymphoproliferation | Fulminant secondary hemophagocytic lymphohistiocytosis with multiorgan involvement requiring specific treatment | Provides an important severe comparator. Unlike the present patient, marked EBV DNAemia was accompanied by systemic EBV disease and multiorgan involvement, illustrating the broad spectrum of post-teplizumab EBV-associated phenotypes |
| **Dias et al., BSPED/ABCD Consensus Statement (10)** | Expert consensus on clinical implementation of teplizumab for stage 2 T1D | Recommends structured pre-treatment safety assessment and monitoring during and after treatment | Recommends assessment for active EBV and CMV infection before teplizumab treatment, with virological findings interpreted in the clinical context | — | Reflects the evolving approach to viral screening and monitoring and supports systematic assessment of EBV in patients receiving teplizumab |

**Abbreviations:** ALT, alanine aminotransferase; AST, aspartate aminotransferase; CMV, cytomegalovirus; CRS, cytokine release syndrome; EBV, Epstein–Barr virus; FDA, Food and Drug Administration; HLH, hemophagocytic lymphohistiocytosis; PCR, polymerase chain reaction; T1D, type 1 diabetes; ULN, upper limit of normal.

**References**

References are self-contained within this Supplementary Table.

1. U.S. Food and Drug Administration. *Multidiscipline Review: TZIELD (teplizumab-mzwv).* Center for Drug Evaluation and Research; 2022.
2. Herold KC, Bundy BN, Long SA, et al. An anti-CD3 antibody, teplizumab, in relatives at risk for type 1 diabetes. *N Engl J Med.* 2019;381:603–613. doi:10.1056/NEJMoa1902226.
3. Herold KC, Gitelman SE, Gottlieb PA, Knecht LA, Raymond R, Ramos EL. Teplizumab: a disease-modifying therapy for type 1 diabetes that preserves β-cell function. Diabetes Care. 2023;46:1848–1856. doi:10.2337/dc23-0675.
4. Ramos EL, Dayan CM, Chatenoud L, et al. Teplizumab and β-cell function in newly diagnosed type 1 diabetes. *N Engl J Med.* 2023;389:2151–2161. doi:10.1056/NEJMoa2308743.
5. European Medicines Agency. *Teplizumab: European Public Assessment Report and Summary of Product Characteristics.* European Medicines Agency; 2026.
6. Gitelman SE, Simmons K, Sherr JL, Leichter SB, Quattrin T, Russell WE, et al. Safety and pharmacokinetics of teplizumab in children less than 8 years of age with stage 2 type 1 diabetes. Diabetologia. (2026) 69:330–42. doi: 10.1007/s00125-025-06586-1.
7. Guarnotta V, Pantò F, Vigneri E, Piombo G, Mineo MI, Tomasello L, Arnaldi G. Teplizumab in stage 2 type 1 diabetes: clinical practice experience on feasibility in 3 adult patients. *Diabetes Obes Metab.* 2026;28(5):4326–4334. doi:10.1111/dom.70626.
8. Karakus KE, Chesshir L, Walker S, Baschal EE, McDaniel KA, Triolo TM, et al. Teplizumab treatment for stage 2 type 1 diabetes: a real-world evaluation of metabolic and immunological outcomes. Diabetologia. 2026;69:1164–1175. doi:10.1007/s00125-025-06646-6.
9. Klemann C, Kordonouri O. Fulminant secondary HLH following EBV reactivation in an adult with trisomy 21 after teplizumab treatment for stage 2 type 1 diabetes. *Diabetes Care.* Published online July 28, 2026. doi:10.2337/dc26-0543.
10. Dias RP, Marcovecchio ML, Willemsen RH, et al. Considerations for the clinical use of teplizumab in stage 2 type 1 diabetes: a Consensus Statement from the British Society of Paediatric Endocrinology and Diabetes (BSPED) and the Association of British Clinical Diabetologists (ABCD). *Diabet Med.* 2026;43:e70329. doi:10.1111/dme.70329.
